# Gene expression profiling in patients with polymyalgia rheumatica before and after symptom-abolishing glucocorticoid treatment

**DOI:** 10.1186/s12891-017-1705-z

**Published:** 2017-08-07

**Authors:** Frederik Flindt Kreiner, Rehannah Borup, Finn Cilius Nielsen, Peter Schjerling, Henrik Galbo

**Affiliations:** 1 Institute for Inflammation Research, Department of Rheumatology Rigshospitalet, Copenhagen University Hospital, Copenhagen, Denmark; 2 Center for Genomic Medicine Rigshospitalet, Copenhagen University Hospital, Copenhagen, Denmark; 3 Institute of Sports Medicine, Department of Orthopedic Surgery M Bispebjerg Hospital and Center for Healthy Aging Faculty of Health and Medical Sciences, University of Copenhagen, Copenhagen, Denmark

**Keywords:** Polymyalgia rheumatica, DNA microarray, Muscle, Gene expression, Prednisolone, Interleukin 6

## Abstract

**Background:**

The pathophysiology, including the impact of gene expression, of polymyalgia rheumatica (PMR) remains elusive. We profiled the gene expression in muscle tissue in PMR patients before and after glucocorticoid treatment.

**Methods:**

Gene expression was measured using Affymetrix Human Genome U133 Plus 2.0 arrays in muscle biopsies from 8 glucocorticoid-naive patients with PMR and 10 controls before and after prednisolone-treatment for 14 days. For 14 genes, quantitative real-time PCR (qRT-PCR, *n* = 9 in both groups) was used to validate the microarray findings and to further investigate the expression of genes of particular interest.

**Results:**

Prednisolone normalized erythrocyte sedimentation rate (ESR) and C-reactive protein (CRP) in PMR patients. A total of 165 putatively clinically relevant, differentially expressed genes were identified (cut-off: fold difference > ±1.2, difference of mean > 30, and *p* < 0.05); of these, 78 genes differed between patients and controls before treatment, 131 genes responded to treatment in a given direction only in patients, and 44 fulfilled both these criteria. In 43 of the 44 genes, treatment counteracted the initial difference. Functional clustering identified themes of biological function, including regulation of protein biosynthesis, and regulation of transcription and of extracellular matrix processes. Overall, qRT-PCR confirmed the microarray findings: Microarray-detected group differences were confirmed for 9 genes in 17 of 18 comparisons (same magnitude and direction of change); lack of group differences in microarray testing was confirmed for 5 genes in 8 of 10 comparisons. Before treatment, using qRT-PCR, expression of interleukin 6 (IL-6) was found to be 4-fold higher in patients (*p* < 0.05).

**Conclusions:**

This study identifies genes in muscle, the expression of which may impact the pathophysiology of PMR. Moreover, the study adds further evidence of the importance of IL-6 in the disease. Follow-up studies are needed to establish the exact pathophysiological relevance of the identified genes.

The study was retrospectively listed on the ISRCTN registry with study ID ISRCTN69503018 and date of registration the 26th of July 2017.

## Background

Polymyalgia rheumatica (PMR) affects men and women above the age of 50 and is recognized as the most common chronic inflammatory, rheumatic disease in this age group [1–3]. Clinically, PMR is associated with prominent muscle complaints, including aching and tender and stiff proximal muscles [1]. Paraclinically, erythrocyte sedimentation rate (ESR) and blood levels of C-reactive protein (CRP) are markedly elevated [1]. Furthermore, concentrations of proinflammatory cytokines, including also interleukin (IL) 6 [4, 5], are elevated systemically as well as locally in muscle tissue [5]. Yet, the prevailing view is that PMR reflects inflammation in the synovia of bursae, joints and tendon sheaths [6]. Overall, however, the current understanding of the etiology, pathogenesis and pathophysiology of PMR is modest. Treatment with glucocorticoids (GCs) is rapidly effective [7, 8], and the majority of patients maintains remission, but many experience at least one GC-related serious adverse event [9].

The genetics of PMR remain elusive; however, the higher incidence in Caucasians [10] and the higher susceptibility in people carrying the HLA-DRB1*04 allele [11] suggest that genetic factors may in fact impact the pathophysiology of the disease. Studies have found associations between polymorphisms in the genes encoding e.g. IL-6 and tumor necrosis factor alpha (TNF-**α**) and the susceptibility to and severity of PMR [12], but generally findings have been inconclusive [13, 14].

In the present study, to extend the understanding of the pathophysiology of PMR, we profiled the gene expression in muscle tissue from GC-naive patients with PMR and matched non-PMR control subjects before and after symptom-eliminating treatment with prednisolone.

## Methods

### Subjects

Nine GC-naive patients with newly diagnosed, untreated PMR and 10 matched (age, sex, and BMI) non-PMR control subjects were studied in the fasting state in the morning before and after 14 days of prednisolone treatment (20 mg/day taken in the morning, also 1–2 h before the second biopsy) in a comprehensive clinical experimental research program, some of the results of which we recently reported [5, 15]. The study was approved by the Ethical Committee of Copenhagen (approval number: KF[01]261665) and informed consent was obtained before study inclusion. Anthropometric data are given in Table 1.

**Table 1:** Characteristics of the PMR patients and the non-PMR control subjects

	PMR patients (*n* = 9)	Controls (*n* = 10)
Female/male	5/4	5/5
Age, mean (range), years	74.2 (60.5–87.2)	72.3 (63.4–85.2)
Body–mass index, mean (range), kg/m^2^	24.3 (16.5–28.7)	25.7 (22.1–29.3)
ESR, mean (range) mm/h		
Before treatment	66 (43–74) ^†^	9 (3–11)
After treatment	13 (4–23) ^‡^	7 (4–10)
CRP, mean (range) mg/l		
Before treatment	55 (27–131)^†^	2 (0–10)
After treatment	5 (0–11)^‡^	2 (1–8)

^†^ *p* < 0.05 vs. control subjects. ^‡^ *p* < 0.05 vs. untreated patients

Patients were diagnosed with PMR according to the criteria proposed by Chuang and colleagues [2, 3, 16, 17], and the diagnosis was later supported by normalization of ESR and CRP upon prednisolone treatment. Patients were recruited by referral from general practitioners; control subjects were recruited by newspaper advertising and included in the study after a standard medical examination and a comprehensive blood and urine screening. Both groups did not meet the exclusion criteria described by Kreiner and colleagues [5]. Controlled chronic comorbidities were accepted in both groups. Diminishing the possibility of occult malignant disease, all subjects had normal thorax X-ray and abdominal ultrasound examination, and negative test for blood in the stools and urine. In addition, all subjects had comprehensive blood screening performed. In patients, only ESR and CRP were different from normal values; no blood values in control subjects were abnormal.

Some subjects received concurrent medication as previously detailed [5]. Before the first experiment, non-steroidal anti-inflammatory drug treatment was not allowed, and use of analgesics was limited to the centrally-acting opioid-like drug tramadol (Mandolgin, Mandoz A/S, Odense, Denmark); none of the subjects had taken tramadol in the morning before any of the two experiments.

### Experiments and interventions

From all subjects, biopsies were obtained from trapezius muscles before and after treatment with prednisolone; in all patients, the trapezius muscle exhibited the symptoms characteristic of PMR, i.e. aching, tenderness and stiffness. Following local anesthesia of the skin and subcutis with Lidocaine (20 mg/mL), muscle tissue was sampled through a small incision in the cutis, subcutis and muscle fascia using a 5 mm Bergström needle with suction [18]. Muscle samples were snap-frozen in liquid nitrogen, weighed (wet weight ranged from 35 to 100 mg per sample), and stored at −80 °C until RNA extraction.

### Total RNA extraction

Total RNA was extracted from 20 to 30 mg muscle sample by tissue homogenization in TriReagent (Molecular Research Center, Cincinnati, Ohio, US) using a bead-mixer (FastPrep®-24 instrument, MP Biomedicals, Illkirch, France) with five inert 2.3 mm steel beads (BioSpec Products, Bartlesville, OK, US) and one siliciumcarbid crystal followed by addition of bromo-chloropropane to separate the homogenate into aqueous and organic phases. To precipitate RNA, isopropanol was added to the isolated aqueous phase. The precipitated total RNA was washed repeatedly in 75% ethanol and dissolved in RNAse-free water before storing at −80 °C until further analysis. Total RNA concentrations were determined by spectroscopy; yields averaged 0.4 μg total RNA/mg muscle tissue.

### DNA microarray analysis

#### Sample preparation and hybridization, and detection and quantification of signals

Total RNA was further purified using RNeasy Mini Kits (Qiagen, Valencia, CA, US), and the integrity and purity of the RNA was verified using an Agilent Bioanalyser (Agilent, Palo Alto, CA, US) as previously described [19]. Based on the quality of the RNA, 8 patient samples and 10 control subject samples were selected for microarray assessment. ds-cDNA was synthesized from 2 μg total RNA using an oligo-dT primer containing a T7 RNA polymerase promoter, and labeled in an T7 promoter-driven in vitro transcription reaction producing biotin-labeled cRNA from the cDNA according to the manufacturer’s (Affymetrix, Santa Clara, CA, US) guidelines. Next, the hybridization mixture was prepared from the fragmented target cRNA as well as probe array controls, bovine serum albumin, and herring sperm DNA.

Affymetrix GeneChip Human Genome U133 Plus 2.0 (Santa Clara, CA, US) arrays, which comprise 54,675 probe sets, were used. Following hybridization, the probe arrays were washed and stained with phycoerytrin streptavidin (SAPE) using the Affymetrix Fluidics Station 450 and scanned using an Affymetrix GeneArray 3000 7G scanner 488 nm to generate fluoresecent images as described in the Affymetrix GeneChip protocol. The amount of bound target at each location of the probe array is proportional to the amount of bound light emitted at 570 nm. Scanned data were stored as image files in cel-format.

#### Data analysis

Cel-files were imported into the statistical software package R v. 2.7.2 using BioConductor v. 2.8 [20], and gcRMA modeled using quantiles normalization and median polish summarization [21]. The modeled log-intensity of approximately 54,600 probe sets was used for selecting differentially expressed genes. The microarray data were submitted to the gene expression repository at Array Express (http://www.ebi.ac.uk/arrayexpress/) with accession number E-MTAB-3671. Differentially expressed genes were selected based on an initial two-way ANOVA analysis including the parameters disease (PMR versus control) and treatment (before versus after treatment) with a *p*-value <0.05 and mutual fold change cut-off of 1.2 and reflecting either main effect or intervention. The resulting 565 selected probe sets were further analyzed. Pairwise differentially expressed transcripts were depicted by a univariate two-sample t-test with equal variance. Multiple testing corrections were performed using the multtest package in Bioconducter v. 2.7.2. Control of Type I error rate was performed by computing adjusted *p*-values for simple multiple testing procedures from a vector of raw (unadjusted) *p*-values by applying the Benjamini & Hochberg FDR analysis [22]. Only transcripts exhibiting a fold change larger than 1.2 and a difference of means larger than 30 (real unlogged values) between (mutual) classes were considered.

#### Gene grouping criteria

Predefined criteria were applied to identify genes of potential pathophysiological impact. The criteria were: 1. difference in expression level between untreated patients and untreated controls (Table 2), and 2. response to prednisolone treatment of expression levels in a given direction in patients only (Table 3). Those genes that differed between untreated patients and controls and that also responded to prednisolone treatment in patients, i.e. the aggregate of criteria 1 and 2, were also identified (criterion 3) (Table 4).

**Table 2:** Genes the expression levels of which differed between untreated patients and untreated controls (78 genes)

Gene symbol	Gene name	Probe set(s)	FD^a^	p
BDNF	brain-derived neurotrophic factor	244503_at	+1.8	0.016
ETS2	v-ets erythroblastosis virus E26 oncogene homolog 2 (avian)	201328_at	+1.8	0.007
SVIP	small VCP/p97-interacting protein	230285_at	+1.7	0.002
SH3RF2	SH3 domain containing ring finger 2	228892_at	+1.6	0.004
TM4SF18	transmembrane 4 L six family member 18	230061_at	+1.5	0.007
TMTC1	transmembrane and tetratricopeptide repeat containing 1	226322_at 226931_at	+1.5 +1.6	0.003 <0.001
TMEM18	transmembrane protein 18	225489_at	+1.5	0.008
N4BP2L1	NEDD4 binding protein 2-like 1	213375_s_at	+1.5	0.019
FMO2	flavin containing monooxygenase 2 (non-functional)	228268_at	+1.5	0.002
RPL37	ribosomal protein L37	224763_at	+1.5	<0.001
CTDSP2	CTD (carboxy-terminal domain. RNA polymerase II. polypeptide A) small phosphatase 2	238999_at	+1.4	0.048
RASL10B	RAS-like. Family 10. member B	235488_at	+1.4	0.012
SMG1P1	nuclear pore complex interacting protein-like	231989_s_at	+1.4	0.008
ZNF331	zinc finger protein 331	219228_at	+1.4	<0.001
FAM184B	family with sequence similarity 184. member B	235288_at	+1.4	0.013
LOC100507303	uncharacterized LOC100507303	228049_x_at	+1.4	0.019
NCKIPSD	NCK interacting protein with SH3 domain	218697_at	+1.4	<0.001
ECHDC3	enoyl CoA hydratase domain containing 3	219298_at	+1.3	0.049
RNF114	ring finger protein 114	200867_at 200868_s_at 211678_s_at	+1.3 +1.3 +1.2	0.006 0.023 0.018
TMPO	thymopoietin	224944_at	+1.3	0.002
RERE	arginine-glutamic acid dipeptide (RE) repeats	200940_s_at	+1.3	0.003
TUBD1	tubulin. Delta 1	231853_at	+1.3	0.003
MARK4	MAP/microtubule affinity-regulating kinase 4	55065_at	+1.3	0.005
ZNF195	zinc finger protein 195	204234_s_at	+1.3	0.003
PCF11	PCF11. cleavage and polyadenylation factor subunit. Homolog (*S. cerevisiae*)	203378_at	+1.3	0.007
DFFA	DNA fragmentation factor. 45 kDa. alpha polypeptide	226116_at	+1.3	0.010
PSPC1	paraspeckle component 1	218371_s_at	+1.3	0.007
RBBP6	retinoblastoma binding protein 6	212783_at	+1.3	0.004
EIF4B	eukaryotic translation initiation factor 4B	211937_at	+1.3	0.017
NPM1	nucleophosmin (nucleolar phosphoprotein B23. numatrin)	221691_x_at	+1.3	0.011
RSBN1	round spermatid basic protein 1	213694_at	+1.2	0.003
PSIP1	PC4 and SFRS1 interacting protein 1	209337_at	+1.2	0.010
EIF3G	eukaryotic translation initiation factor 3. subunit G	208887_at	+1.2	0.006
COL4A3BP	collagen. Type IV. alpha 3 (Goodpasture antigen) binding protein	219625_s_at 223465_at	+1.2 +1.2	0.003 0.029
PCID2	PCI domain containing 2	219940_s_at	+1.2	0.003
PXDC1	PX domain containing 1	212923_s_at	+1.2	0.042
BCKDHA	branched chain keto acid dehydrogenase E1, alpha polypeptide	202331_at	+1.2	0.024
AKR7A2	aldo-keto reductase family 7, member A2	202139_at	+1.2	0.010
MRPS2	mitochondrial ribosomal protein S2	218001_at	+1.2	0.018
RORA	RAR-related orphan receptor A	226682_at	+1.2	0.049
RPL36AL	ribosomal protein L36a-like	207585_s_at	+1.2	0.011
TFRC	transferrin receptor (p90, CD71)	208691_at	–3.0	0.004
SFRP4	secreted frizzled-related protein 4	204051_s_at 204052_s_at	−2.9	0.001 0.002
NOV	nephroblastoma overexpressed	214321_at	−2.0	0.037
PAQR9	progestin and adipoQ receptor family member IX	1558322_a_at	−2.0	<0.001
C2orf88	chromosome 2 open reading frame 88	228195_at	−1.9	0.011
FAM69A	family with sequence similarity 69, member A	213689_x_at	−1.8	0.001
TP53INP2	tumor protein p53 inducible nuclear protein 2	224836_at	−1.8 −1.9	<0.001 0.002
SH3KBP1	SH3-domain kinase binding protein 1	1554168_a_at 223082_at	−1.8	0.002
NINJ2	ninjurin 2	219594_at	−1.7	0.039
MEST	mesoderm specific transcript homolog (mouse)	202016_at	−1.7	0.010
ITGB1BP2	integrin beta 1 binding protein (melusin) 2	219829_at	−1.6	<0.001
PLXDC1	plexin domain containing 1	219700_at	−1.5	0.006
BPGM	2,3-bisphosphoglycerate mutase	203502_at	−1.5	<0.001
MTFP1	mitochondrial fission process 1	223172_s_at	−1.5	0.004
MAP2K3	mitogen-activated protein kinase kinase 3	215499_at	−1.5	0.003
LRRN4CL	LRRN4 C-terminal like	1556427_s_at	−1.4	0.042
FBXO9	F-box protein 9	210638_s_at212987_at	−1.4 −1.4	<0.001 <0.001
HERC1	HECT and RLD domain containing E3 ubiquitin protein ligase family member 1	218306_s_at	−1.4	<0.001
JARID2	jumonji, AT rich interactive domain 2	203297_s_at	−1.4	<0.001
TRAK1	trafficking protein, kinesin binding 1	202079_s_at	−1.4	0.004
ZNF252P	zinc finger protein 252, pseudogene	228200_at	−1.4	<0.001
PRSS23	protease, serine, 23	202458_at	−1.4	0.030
OLFML2B	olfactomedin-like 2B	213125_at	−1.4	0.049
MSANTD4	Myb/SANT-like DNA-binding domain containing 4 with coiled-coils	227418_at	−1.3	0.043
ZDHHC7	zinc finger, DHHC-type containing 7	218606_at	−1.3	<0.001
RAP2A	RAP2A, member of RAS oncogene family	225585_at	−1.3	0.016
LRP12	low density lipoprotein receptor-related protein 12	219631_at	−1.3	0.050
BMPR1A	bone morphogenetic protein receptor, type IA	213578_at	−1.3	0.001
RNF10	ring finger protein 10	207801_s_at	−1.3	<0.001
COL5A1	collagen, type V, alpha 1	203325_s_at	−1.3	0.007
INSIG1	insulin induced gene 1	201626_at	−1.3	0.046
SLC35E3	solute carrier family 35, member E3	218988_at	−1.3	0.003
MEMO1	dpy-30 homolog (*C. elegans*) /// mediator of cell motility 1	219065_s_at	−1.3	0.004
MYL4	myosin, light chain 4, alkali; atrial, embryonic	210395_x_at	−1.2	0.002
COX7A2	cytochrome c oxidase subunit VIIa polypeptide 2 (liver)	201597_at	−1.2	0.019
MGAT4B	mannosyl (alpha-1,3-)-glycoprotein beta-1,4-N-acetylglucosaminyltransferase, isozyme B	224598_at	−1.2	0.003
MRC2	mannose receptor, C type 2	209280_at	−1.2	0.010

*FD* fold difference. ^a^ fold differences for genes with more than one probe set were calculated as the average of the individual values, which did not differ markedly

**Table 3:** Genes the expression levels of which responded to prednisolone treatment in a given direction only in patients with polymyalgia rheumatica (131 genes)

Gene symbol	Gene name	Probe set(s)	FC^a^	p
COL1A1	collagen, type I, alpha 1	1556499_s_at	+4.7	0.028
CTGF	connective tissue growth factor	209101_at	+2.9	0.012
MEST	mesoderm specific transcript homolog (mouse)	202016_at	+2.7	0.049
CDH11	cadherin 11, type 2, OB-cadherin (osteoblast)	207173_x_at	+2.6	0.012
S1PR3	sphingosine-1-phosphate receptor 3	228176_at	+2.5	0.009
CD248	CD248 molecule, endosialin	219025_at	+2.5	0.019
FBN1	fibrillin 1	202766_s_at 235318_at	+2.4 +2.1	0.031 0.017
NINJ2	ninjurin 2	219594_at	+2.3	0.002
MFAP5	microfibrillar associated protein 5	209758_s_at 213764_s_at 213765_at	+2.7 +2.2 +2.1	0.038 0.010 0.018
SH3PXD2B	SH3 and PX domains 2B	231823_s_at	+2.2	0.011
C13orf33	chromosome 13 open reading frame 33	227058_at	+2.2	0.044
FOSL2	FOS-like antigen 2	218880_at	+2.2	0.026
BGN	biglycan	201261_x_at	+2.1	0.029
NEDD9	neural precursor cell expressed, developmentally down-regulated 9	233223_at	+2.1	0.004
COL5A2	collagen, type V, alpha 2	221730_at	+2.0	0.049
NT5E	5′-nucleotidase, ecto (CD73)	203939_at	+2.0	0.044
TUBB6	tubulin, beta 6 class V	209191_at	+2.0	0.031
SPARC	secreted protein, acidic, cysteine-rich (osteonectin)	200665_s_at	+2.0	0.043
FN1	fibronectin 1	210495_x_at 211719_x_at 212464_s_at 216442_x_at	+1.9 +1.9 +1.9 +2.0	0.045 0.042 0.046 0.038
GFPT2	glutamine-fructose-6-phosphate transaminase 2	205100_at	+1.9	0.034
NFKBIZ	nuclear factor of kappa light polypeptide gene enhancer in B-cells inhibitor, zeta	223217_s_at	+1.9	0.025
DCLK1	doublecortin-like kinase 1	205399_at	+1.9	0.034
METRNL	meteorin, glial cell differentiation regulator-like	225955_at	+1.9	0.023
COL1A2	collagen, type I, alpha 2	229218_at	+1.8	0.048
LAMB1	laminin, beta 1	201505_at	+1.8	0.003
LSP1P1	lymphocyte-specific protein 1 pseudogene	214110_s_at	+1.8	0.020
COL6A3	collagen, type VI, alpha 3	201438_at	+1.8	0.003
GAS7	growth arrest-specific 7	202191_s_at 202192_s_at	+1.8 +1.7	0.028 0.021
ARHGAP26	Rho GTPase activating protein 26	244548_at	+1.8	0.003
OLFML2B	olfactomedin-like 2B	213125_at	+1.7	0.031
SPON2	spondin 2, extracellular matrix protein	218638_s_at	+1.7	0.002
COL6A1	collagen, type VI, alpha 1	213428_s_at	+1.7	0.006
CILP	cartilage intermediate layer protein, nucleotide pyrophosphohydrolase	206227_at	+1.7	0.012
OLFML3	olfactomedin-like 3	218162_at	+1.7	0.026
FAM69A	family with sequence similarity 69, member A	213689_x_at	+1.7	<0.001
CORO1C	coronin, actin binding protein, 1C	222409_at	+1.6	0.020
MAP1B	microtubule-associated protein 1B	226084_at	+1.6	0.039
COL6A2	collagen, type VI, alpha 2	209156_s_at	+1.6	0.020
PRKCDBP	protein kinase C, delta binding protein	213010_at	+1.6	<0.001
CLIC4	chloride intracellular channel 4	201560_at	+1.6	0.010
LRRN4CL	LRRN4 C-terminal like	1556427_s_at	+1.5	0.006
CD109	CD109 molecule	226545_at	+1.5	0.034
DBN1	drebrin 1	202806_at	+1.5	0.020
SFXN3	sideroflexin 3	220974_x_at	+1.5	0.016
TNXA / TNXB	**tenascin XA (pseudogene) / tenascin XB**	**206093_x_at** **213451_x_at** **216333_x_at**	**+1.5** **+1.5** **+1.5**	**0.030** **0.034** **0.041**
PRSS23	protease, serine, 23	202458_at	+1.5	0.022
TUBA1A	tubulin, alpha 1a	209118_s_at	+1.5	0.038
SAMHD1	SAM domain and HD domain 1	235529_x_at	+1.5	0.024
ITGB1BP2	integrin beta 1 binding protein (melusin) 2	219829_at	+1.5	0.003
ATP2C1	ATPase, Ca++ transporting, type 2C, member 1	209934_s_at	+1.5	<0.001
PXDC1	PX domain containing 1	212923_s_at	+1.5	0.014
PAQR9	progestin and adipoQ receptor family member IX	1558322_a_at	+1.4	0.027
P4HA2	prolyl 4-hydroxylase, alpha polypeptide II	202733_at	+1.4	0.024
ANXA2	annexin A2	201590_x_at 210427_x_at 213503_x_at	+1.4 +1.4 +1.4	0.025 0.027 0.032
ACVRL1	activin A receptor type II-like 1	226950_at	+1.4	0.009
CHSY1	chondroitin sulfate synthase 1	203044_at	+1.4	0.021
C10orf54	chromosome 10 open reading frame 54	225373_at	+1.4	0.016
PLAGL1	pleiomorphic adenoma gene-like 1	207943_x_at	+1.4	0.012
CTTNBP2NL	CTTNBP2 N-terminal like	226000_at	+1.4	0.019
SYNPO2	synaptopodin 2	225720_at	+1.4	0.013
ANXA2P2	annexin A2 pseudogene 2	208816_x_at	+1.4	0.042
TGFB1I1	transforming growth factor beta 1 induced transcript 1	209651_at	+1.4	0.043
ACTB	actin, beta	213867_x_at 224594_x_at 200801_x_at	+1.4 +1.4 +1.4	0.048 0.040 0.033
TRIO	triple functional domain (PTPRF interacting)	208178_x_at 209012_at	+1.4	0.018
ITGA5	integrin, alpha 5 (fibronectin receptor, alpha polypeptide)	201389_at	+1.4	0.038
RRBP1	ribosome binding protein 1 homolog 180 kDa (dog)	201204_s_at	+1.4	0.010
LASP1	LIM and SH3 protein 1	200618_at	+1.4	0.016
ADNP2	ADNP homeobox 2	203321_s_at	+1.3	0.009
MTFP1	mitochondrial fission process 1	223172_s_at	+1.3	0.017
TP53INP2	tumor protein p53 inducible nuclear protein 2	224836_at	+1.3	0.017
PDGFRB	platelet-derived growth factor receptor, beta polypeptide	202273_at	+1.3	0.009
FBXO9	F-box protein 9	210638_s_at 212987_at	+1.3 +1.3	0.002 <0.001
VAT1	vesicle amine transport protein 1 homolog (*T. californica*)	208626_s_at	+1.3	0.043
LTBP1	latent transforming growth factor beta binding protein 1	202729_s_at	+1.3	0.026
HIF1A	hypoxia inducible factor 1, alpha subunit	200989_at	+1.3	0.025
SH3KBP1	SH3-domain kinase binding protein 1	1554168_a_at 223082_at	+1.3 +1.3	0.044 0.027
JARID2	jumonji, AT rich interactive domain 2	203297_s_at	+1.3	0.007
ACTG1	actin, gamma 1	201550_x_at 211970_x_at 211983_x_at 211995_x_at 212363_x_at 212988_x_at 213214_x_at	+1.3 +1.3 +1.3 +1.3 +1.3 +1.3 +1.3	0.015 0.009 0.031 0.013 0.020 0.017 0.021
MAP2K3	mitogen-activated protein kinase kinase 3	215499_at	+1.3	0.021
MEMO1	mediator of cell motility 1	219065_s_at	+1.3	0.012
EZR	ezrin	208623_s_at	+1.3	0.002
BPGM	2,3-bisphosphoglycerate mutase	203502_at	+1.2	0.036
TUBB	tubulin, beta class I	212320_at	+1.2	0.039
DDAH1	dimethylarginine dimethylaminohydrolase 1	209094_at	+1.2	0.033
BDNF	brain-derived neurotrophic factor	244503_at	−3.1	0.001
SLC25A34	solute carrier family 25, member 34	1559977_a_at 232245_at	−1.9 −1.9	0.006 0.009
SVIP	small VCP/p97-interacting protein	230285_at	−1.7	0.004
VPS8	vacuolar protein sorting 8 homolog (S. cerevisiae)	239917_at	−1.6	<0.001
PIAS2	protein inhibitor of activated STAT, 2	244633_at	−1.6	0.011
LOC100507303	uncharacterized LOC100507303	228049_x_at	−1.6	0.004
RPL37	ribosomal protein L37	224763_at	−1.5	<0.001
TMTC1	transmembrane and tetratricopeptide repeat containing 1	226322_at 226931_at	−1.4 −1.6	0.005 <0.001
MLYCD	malonyl-CoA decarboxylase	218869_at	−1.5	0.004
UCP3	uncoupling protein 3 (mitochondrial, proton carrier)	207349_s_at	−1.5	0.016
TUBD1	tubulin, delta 1	231853_at	−1.4	0.003
BCKDHA	branched chain keto acid dehydrogenase E1, alpha polypeptide	202331_at	−1.4	0.004
TRIM39	tripartite motif containing 39	222732_at	−1.4	0.002
ZNF331	zinc finger protein 331	219228_at	−1.4	0.003
NRBF2	nuclear receptor binding factor 2	223650_s_at	−1.4	0.021
GTF2H5	general transcription factor IIH, polypeptide 5	244294_at	−1.4	0.007
FMO2	flavin containing monooxygenase 2 (non-functional)	228268_at	−1.4	0.002
TMEM18	transmembrane protein 18	225489_at	−1.4	0.028
HSDL2	Hydroxysteroid dehydrogenase like 2	215436_at	−1.4	0.006
N4BP2L1	NEDD4 binding protein 2-like 1	213375_s_at	−1.4	0.033
PEBP4	phosphatidylethanolamine-binding protein 4	227848_at	−1.4	0.009
RANBP9	RAN binding protein 9	216125_s_at	−1.4	0.002
ST3GAL5	ST3 beta-galactoside alpha-2,3-sialyltransferase 5	203217_s_at	−1.3	0.003
ACADSB	acyl-CoA dehydrogenase, short/branched chain	226030_at	−1.3	0.006
RNF114	ring finger protein 114	200867_at 200868_s_at 211678_s_at	−1.3 −1.3 −1.2	0.020 0.030 0.041
MRPS2	mitochondrial ribosomal protein S2	218001_at	−1.3	0.006
TMEM50B	transmembrane protein 50B	219600_s_at	−1.3	0.027
EIF3G	eukaryotic translation initiation factor 3, subunit G	208887_at	−1.3	0.005
PSIP1	PC4 and SFRS1 interacting protein 1	209337_at	−1.3	0.007
PTP4A1	protein tyrosine phosphatase type IVA, member 1	200732_s_at	−1.3	<0.001
EIF4B	eukaryotic translation initiation factor 4B	211937_at	−1.3	0.015
FAM184B	family with sequence similarity 184, member B	235288_at	−1.3	0.042
CNNM3	cyclin M3	229031_at	−1.3	0.011
RERE	arginine-glutamic acid dipeptide (RE) repeats	200940_s_at	−1.3	0.008
ZNF195	zinc finger protein 195	204234_s_at	−1.3	0.002
SNRPA	small nuclear ribonucleoprotein polypeptide A	201770_at	−1.3	0.025
TM4SF18	transmembrane 4 L six family member 18	230061_at	−1.3	0.033
RPL36AL	ribosomal protein L36a-like	207585_s_at	−1.2	0.008
RBBP6	retinoblastoma binding protein 6	212783_at	−1.2	0.025
TSFM	Ts translation elongation factor, mitochondrial	214331_at	−1.2	0.019
POLR1B	polymerase (RNA) I polypeptide B, 128 kDa	223403_s_at	−1.2	0.018
NPM1	nucleophosmin (nucleolar phosphoprotein B23, numatrin)	221691_x_at	−1.2	0.022
OXA1L	oxidase (cytochrome c) assembly 1-like	208717_at	−1.2	0.027
RSBN1	round spermatid basic protein 1	213694_at	−1.2	0.016
AKR7A2	aldo-keto reductase family 7, member A2	202139_at	−1.2	0.002
RORA	**RAR-related orphan receptor A**	**226682_at**	**−1.2**	**0.044**
DFFA	DNA fragmentation factor, 45 kDa, alpha polypeptide	226116_at	−1.2	0.016

FC, fold change. Entries in **bold** indicate that genes also responded significantly (but in the opposite direction) in control subjects. Responses in controls for both these genes, TNXA/TNXB and RORA, were of the same magnitude as in patients but in the opposite direction. ^a^fold changes for genes with more than one probe set were calculated as the average of the individual values, which did not differ markedly

**Table 4:** Genes the expression levels of which differed between untreated patients with polymyalgia rheumaticaand untreated controls (FD), and which responded to prednisolone treatment in the patients (FC) (44 genes)

Gene symbol	Gene name	FD^a^	p	FC^b^	p
BDNF	brain-derived neurotrophic factor	+1.8	0.016	−3.1	0.001
SVIP	small VCP/p97-interacting protein	+1.7	0.002	−1.7	0.004
TM4SF18	transmembrane 4 L six family member 18	+1.5	0.007	−1.3	0.033
TMTC1	transmembrane and tetratricopeptide repeat containing 1	+1.5	0.001	−1.5	0.003
TMEM18	transmembrane protein 18	+1.5	0.008	−1.4	0.028
N4BP2L1	NEDD4 binding protein 2-like 1	+1.5	0.019	−1.4	0.033
FMO2	flavin containing monooxygenase 2 (non-functional)	+1.5	0.002	−1.4	0.012
RPL37	ribosomal protein L37	+1.5	<0.001	−1.5	<0.001
FAM184B	family with sequence similarity 184, member B	+1.4	0.013	−1.3	0.042
LOC100507303	uncharacterized LOC100507303	+1.4	0.019	−1.6	0.004
RNF114	ring finger protein 114	+1.3	0.016	−1.3	0.030
RERE	arginine-glutamic acid dipeptide (RE) repeats	+1.3	0.003	−1.3	0.008
TUBD1	tubulin, delta 1	+1.3	0.003	−1.4	0.003
ZNF195	zinc finger protein 195	+1.3	0.003	−1.3	0.002
DFFA	DNA fragmentation factor, 45 kDa, alpha polypeptide	+1.3	0.010	−1.2	0.016
RBBP6	retinoblastoma binding protein 6	+1.3	0.004	−1.2	0.025
NPM1	nucleophosmin (nucleolar phosphoprotein B23, numatrin)	+1.3	0.011	−1.2	0.022
EIF4B	eukaryotic translation initiation factor 4B	+1.3	0.017	−1.3	0.015
RSBN1	round spermatid basic protein 1	+1.2	0.003	−1.2	0.016
PSIP1	PC4 and SFRS1 interacting protein 1	+1.2	0.010	−1.3	0.007
EIF3G	eukaryotic translation initiation factor 3, subunit G	+1.2	0.006	−1.3	0.005
PXDC1	PX domain containing 1	+1.2	0.042	+1.5	0.014
BCKDHA	branched chain keto acid dehydrogenase E1, alpha polypeptide	+1.2	0.024	−1.4	0.004
AKR7A2	aldo-keto reductase family 7, member A2	+1.2	0.010	−1.2	0.002
MRPS2	mitochondrial ribosomal protein S2	+1.2	0.018	−1.3	0.006
RORA	**RAR-related orphan receptor A**	**+1.2**	**0.049**	**−1.2**	**0.044**
RPL36AL	ribosomal protein L36a-like	+1.2	0.011	−1.2	0.008
PAQR9	progestin and adipoQ receptor family member IX	−2.0	<0.001	+1.4	0.027
FAM69A	family with sequence similarity 69, member A	−1.8	0.001	+1.7	<0.001
TP53INP2	tumor protein p53 inducible nuclear protein 2	−1.8	<0.001	+1.3	0.017
SH3KBP1	SH3-domain kinase binding protein 1	−1.8	0.002	+1.3	0.035
NINJ2	ninjurin 2	−1.7	0.039	+2.3	0.002
MEST	mesoderm specific transcript homolog (mouse)	−1.7	0.010	+2.7	0.049
ITGB1BP2	integrin beta 1 binding protein (melusin) 2	−1.6	<0.001	+1.5	0.003
BPGM	2,3-bisphosphoglycerate mutase	−1.5	<0.001	+1.2	0.036
MTFP1	mitochondrial fission process 1	−1.5	0.004	+1.3	0.017
MAP2K3	mitogen-activated protein kinase kinase 3	−1.5	0.003	+1.3	0.021
LRRN4CL	LRRN4 C-terminal like	−1.4	0.042	+1.5	0.006
FBXO9	F-box protein 9	−1.4	<0.001	+1.3	0.001
JARID2	jumonji, AT rich interactive domain 2	−1.4	<0.001	+1.3	0.007
PRSS23	protease, serine, 23	−1.4	0.030	+1.5	0.022
OLFML2B	olfactomedin-like 2B	−1.4	0.049	+1.7	0.031
MEMO1	mediator of cell motility 1	−1.3	0.004	+1.3	0.012

*FD* fold difference, *FC* fold change

^a^ + and −; expression levels were higher and lower, respectively, in patients with polymyalgia rheumatica than in controls before treatment with prednisolone

^b^ + and −; expression levels increased and decreased, respectively, in patients with polymyalgia rheumatica after treatment with prednisolone

Entry in **bold** indicates that the gene also responded significantly to prednisolone in controls. The response in controls for the RORA gene was of the same magnitude as in patients but in the opposite direction

#### Assessment of biological function

For genes in all three criteria sets, biological functions were assessed using the Database for Annotation, Visualization and Integrated Discovery (DAVID) tool [23] with default options and annotations current as of February 2013. Functional annotation clustering was performed; this process associates individual genes in a large gene list with biological terms and group sets of genes according to functionally similar terms. Moreover, the importance of each cluster is ranked using enrichment scores, which are the geometric means of the enrichment *P* values (EASE score [24]) for each annotation term in the cluster. While enrichment scores above 1.3 are considered particularly interesting, clusters with scores below 1.3 could also be of central importance (e.g. short gene lists do not generally get very high enrichment scores, illustrating that categories with lower scores may still be biologically relevant) [23]. In the presentation of the results, clusters with the highest enrichment scores will be presented.

### Quantitative RT-PCR

To confirm mRNA level fold differences and fold changes found using the microarrays, mRNA levels for a selection (Tables 5 and 6) of the filtered genes were measured using quantitative real-time PCR (qRT-PCR). Moreover, mRNA levels for additional genes (Table 5) that did not differ using microarrays, but which were of particular interest in elucidating the PMR disease mechanisms, were included in the qRT-PCR analysis.

**Table 5:** Quantitative RT-PCR fold differences between untreated patients with polymyalgia rheumatica (PMR) and non-PMR controls, and fold changes between treated and untreated PMR patients

Gene symbol	Fold differences^b^		Fold changes^c^	
(probe name)	qRT-PCR	Microarray^a^	qRT-PCR	Microarray^**a**^
*Genes that differed in microarray testing in at least one comparison*				
BDNF	+1.90*	+1.80 *	−1.58 **	−3.1 **
COL5A1	−1.33 ns	−1.30 **	+1.73 ns	+2.30 ns
EIF4B	+1.63 *p* = 0.0504	+1.30 *	−1.23 *	−1.30 *
MARK4	+1.32 ns	+1.30 **	−1.24 *	−1.15 ns
MTFP1	+1.00 ns	−1.50 **	+1.33 *	+1.30 *
NPM1	+1.38 **	+1.30 *	−1.09 ns	−1.22 *
PRSS23	−1.21 ns	−1.40 *	+1.27 ns	+1.51 *
TFRC	−1.63 ns	−3.00 *	+1.17 ns	+1.76 ns
TUBD1	+1.26 **	+1.30 **	−1.08 ns	−1.40 **
*Genes that did not differ in microarray testing*				
ACTA1 (203872_at)	−1.03 ns	−1.02 ns	1.06 ns	+1.00 ns
DES^a^ (216947_at 202222_s_at 214027_x_at)	+1.16 ns	+1.00 ns	−1.07 ns	+1.00 ns
IL6 (205207_at)	+4.54 *	+1.02 ns	−3.25 *	+1.02 ns
TNFA (207113_s_at)	+1.31 ns	+1.00 ns	−1.31 ns	−1.00 ns
TUBA8 (220069_at)	−1.02 ns	−1.02 ns	+1.10 ns	+1.00 ns

*qRT-PCR* quantitative real-time PCR

^*^ *p* < 0.05. ^**^ *p* < 0.01. ns, not statistically significant. Data are geometric means

^a^ Microarray numbers were calculated as the mean of the individual probe values

^b^ + and −, expression levels were higher and lower, respectively, in patients with polymyalgia rheumatica than in controls before treatment with prednisolone

^c^ + and −, expression levels increased and decreased, respectively, in patients with polymyalgia rheumatica after treatment with prednisolone

**Table 6:** qRT-PCR primer sequences

Gene	Sense	Antisense
ACTA1	GCCGTGTTCCCGTCCATCGT	TTCAGGGTCAGGATACCTCTCTTGCT
BDNF	GAGGGGAGCTGAGCGTGTGTG	TTTTTGTCTGCCGCCGTTACCC
COL5A1	CGCCGACACCTCCAACTCCTC	CTCAGTGAACTCCCCCTCCAA
DES	CCATCCAGACCTACTCTGCCCTC	TTGGTATGGACCTCAGAACCCCTTT
EIF4B	CGTCAGCTGGATGAGCCAAAA	GTCCTCGACCGTTCCCGTTCC
IL6	GAGGCACTGGCAGAAAACAACC	CCTCAAACTCCAAAAGACCAGTGATG
MARK4	AGATCCCAGAGCGGCGGAAG	GGGTCATCATGCTAGGAGGGAGGTT
MTFP1	AAGGCAAGAAGGCTGGAGAGGTG	ACAGAGGCTAGAGCCTGCCATACAAA
NPM1	GGTTTCCCTTGGGGGCTTTG	GCACTGGCCCTGAACCACACTT
PRSS23	CAGCGGGTCTGGGGTCTATG	GCCAATAATTTTTCGCTCCCACTTCT
TUBD1	TGATTGTTGGGAAGGCATGGA	CAACAACCTGCTCTAATGACGTGAAA
TFRC	TCGGGAATGCTGAGAAAACAGACA	TTTTGGAGATACGTAGGGAGAGAGGAA
TNFA	TTCCCCAGGGACCTCTCTCTAATC	GAGGGTTTGCTACAACATGGGCTAC
TUBA8	GCCCAAGGATGTGAATGTCGCT	GGTCGGGGGCTGGTAGTTGATG
RPLP0	GGAAACTCTGCATTCTCGCTTCCT	CCAGGACTCGTTTGTACCCGTTG

*qRT-PCR* quantitative real-time PCR

The primer set sequence for BDNF provided in Table 6 recognizes all BDNF isoforms; using this primer set, the results presented in Table 5 were obtained. The BDNF mRNA levels were also assessed with qRT-PCR using a BDNF primer set that specifically recognizes the BDNF isoform that is recognized by the probe on the used microarray; the results (fold difference + 1.53, *p* < 0.1; fold change −2.4, *p* < 0.01) from this additional assessment were very similar to the results presented in Table 5

From 9 patient samples and 9 control subject samples, cDNA was synthesized using Omniscript reverse transcriptase (Qiagen, Hilden, Germany) from 500 ng total RNA (same pool as used in the microarray runs) in 20 μl. For each target mRNA, 0.25 μl cDNA was amplified in 25 μl Quantitect SYBR Green Master Mix (Qiagen) with corresponding primers (100 nM of both antisense and sense primers, Table 6) on a Stratagene MX3000P RT-PCR instrument (Stratagene, La Jolla, CA, US).

The applied thermal profile was as follows: 95°Celsius, 10 min-(95 °C, 15 s-58 °C, 30s-63°C, 90s)×50–95 °C, 60s-55°C, 30s-95°C, 60s. Standard curves were made using dilution series of a cDNA pool and related to the threshold cycles (C_t_) at the 63 °C step at which the signal intensity was acquired. To ensure specificity, melting curves were analyzed post amplification (at the 55 °C to 95 °C step). The C_t_ values for the samples were converted to relative values using the standard curves and normalized to the internal “housekeeping” control, ribosomal protein P0 (RPLP0). Microarray analysis confirmed that the RPLP0 mRNA level is stable under the current conditions and therefore suitable as the normalizer.

### Statistics

Statistical methods used in the evaluation of the microarray data are described above. Data are reported in compliance with the guidelines for minimum information about a microarray experiment (MIAME).

Statistical analyses of qRT-PCR and anthropometric data as well as of ESR and CRP levels were performed using SPSS software version 20.0 for Macintosh. qRT-PCR data were log-transformed. Statistically significant differences were detected using Student’s t tests, paired or unpaired as applicable. Identical conclusions were achieved with standard non-parametric tests. *P*-values less than 0.05 were considered significant in two-tailed testing.

## Results

Clinical characteristics for all participants are given in Table 1. In all of the PMR patients, treatment with prednisolone abolished symptoms within a few days, supporting the PMR diagnosis; at day 15, ESR and CRP levels were markedly reduced in the patients and did no longer differ significantly from values in controls (Table 1). Control subjects had normal ESR and CRP values both before and after treatment (Table 1).

### Differential expression of genes in untreated PMR patients vs controls

565 transcripts were differentially expressed between patients and controls or before vs after treatment with prednisolone, reflecting either main effect or interaction. Among these transcripts, 165 genes fulfilled at least one of the 2 criteria (Methods) that define the potentially, clinically relevant genes.

**Fig. 1:**
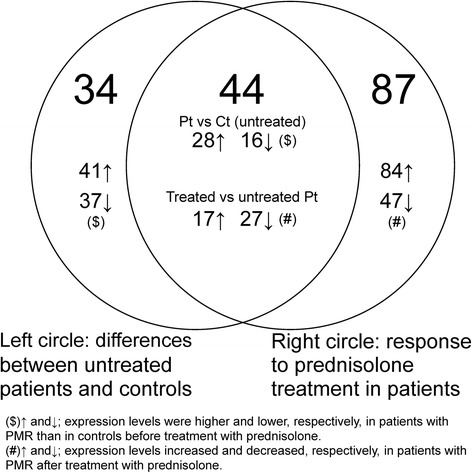
Venn-diagram showing 1. the number of genes that differed between untreated patients with polymyalgia rheumatica (PMR) and non-PMR controls (left circle, 34 + 44 genes) and 2. the number of genes that responded to treatment with prednisolone in a given direction in patients with PMR only (right circle, 44 + 87 genes). The overlap of the two circles includes the number of genes which fulfilled both criteria 1 and 2 (44)

Of the 165 genes, expression levels of 78 genes differed between patients and controls before treatment (Fig. 1, Table 2). Among these genes, 41 genes were upregulated in the patients (mean fold difference: 1.4; range: 1.2–1.8), while 37 were downregulated (mean fold difference: 1.5; range: 1.2 − 3.0).

**Fig. 2:**
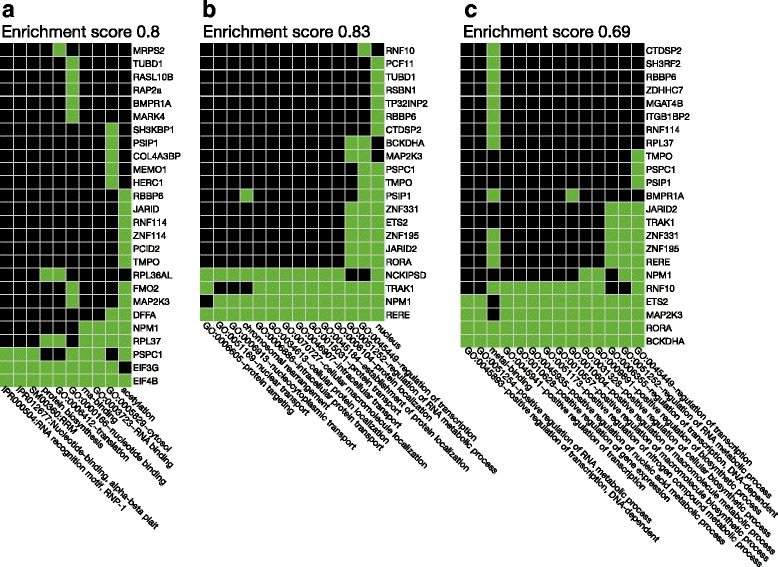
Selected clusters of similar biologic functional terms for genes, the expression of which differed between untreated patients with polymyalgia rheumatica (PMR) and non-PMR control subjects. The clusters and the enrichment scores (the geometric means of the EASE scores [24] of all terms in the cluster) were derived using the Database for Annotation, Visualization and Integrated Discovery (DAVID) tool [23]. Green squares denote that the gene/term association has been positively reported; black squares denote that the gene/term association has not yet been reported. **a** Cluster with an overall theme of translation/protein biosynthesis and with an enrichment score of 0.8. **b** Cluster with an overall theme of (nuclear) protein transport associated processes and with an enrichment score of 0.83. **c** Cluster with an overall theme of gene expression/transcription regulatory processes and with an enrichment score of 0.69

In this subset, the biological function (Fig. 2) of the 78 genes as identified by the DAVID functional annotation clusters (19 clusters in total) included translation/protein biosynthesis (2 clusters, enrichment scores 0.8 and 0.62 [data not shown), transcription/regulation of transcription (2 clusters, enrichment score 0.69 and 0.4 [data not shown), nuclear transport and protein transport (enrichment score 0.83), and SH3 domain binding properties (enrichment score 1.15 [data not shown).

### Genes responding to prednisolone in PMR patients

**Fig. 3:**
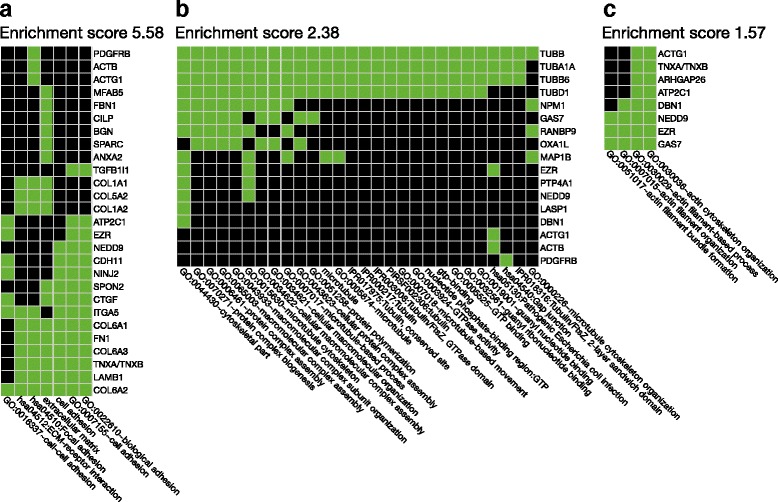
Selected clusters of similar biologic functional terms for genes, the expression of which responded to treatment with prednisolone in a given direction only in patients with polymyalgia rheumatica (PMR); two of the genes in this group of genes (*n* = 131) also responded in non-PMR controls, but in the opposite direction to that seen in patients. The clusters and the enrichment scores (the geometric means of the EASE scores [24] of all terms in the cluster) were derived using the Database for Annotation, Visualization and Integrated Discovery (DAVID) tool [23]. Green squares denote that the gene/term association has been positively reported; black squares denote that the gene/term association has not yet been reported. **a** Cluster with an overall theme of extracellular matrix/cell adhesion processes and with an enrichment score of 5.58. **b** Cluster with an overall theme of cytoskeleton/microtubule associated processes and with an enrichment score of 2.38. **c** Cluster with an overall theme of cytoskeleton/actin filament associated processes and with an enrichment score of 1.57

Expression of 131 of the total 165 genes responded to prednisolone treatment in patients (Fig. 1 and Table 3); of these genes, two responded significantly to treatment in controls, however in the opposite direction to that seen in patients. Of the 131 genes, the expression of 84 genes was up-regulated upon treatment (mean fold change: 1.7; range: 1.2–4.7); 47 genes were down-regulated (mean fold difference: 1.4; range: 1.2–3.1). In this subset, out of a total of 62 DAVID-identified clusters, the clusters of interesting biological function and high enrichment scores (Fig. 3) included extracellular matrix organization and cell adhesion (2 highly enriched clusters, enrichment scores 5.58 and 4.11 [not shown in Fig. 3]), cytoskeleton/microtubule organization (2 clusters, enrichment scores 2.38 and 1.62 [not shown in Fig. 3]), and actin filament/cytoskeleton associated processes (1 cluster, enrichment score 1.57).

### Genes differentially expressed in untreated PMR patients vs controls and also responding to prednisolone in patients

Among all 165 differentially expressed genes were 44 genes, the expression levels of which differed between untreated patients and controls and which in patients only also responded to prednisolone treatment in a given direction (Fig. 1 and Table 4). Of these 44 genes, the expression levels of 28 genes were higher in untreated patients than in untreated controls (mean fold difference: 1.4; range: 1.2–1.8); the expression levels of 16 genes were lower (mean fold difference: 1.4; range: 1.2–2.0). Upon prednisolone treatment, the expression levels of 27 were down-regulated in patients (mean fold change: 1.4; range: 1.2–3.1), whereas 17 genes were up-regulated (mean fold change: 1.5; range: 1.2–2.7). None of the 44 genes responded significantly to prednisolone treatment in control subjects.

**Fig. 4:**
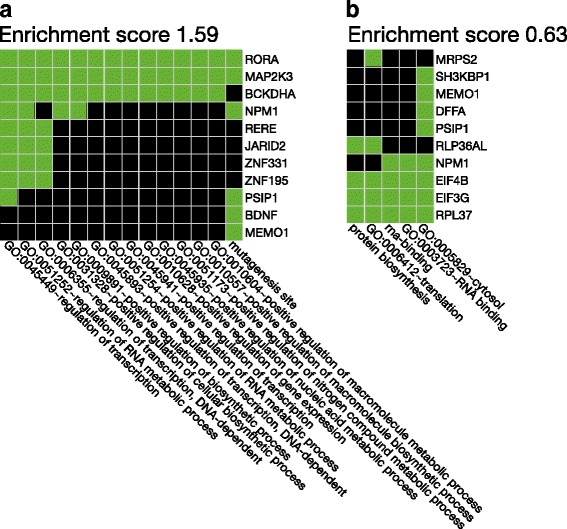
Selected clusters of similar biologic functional terms for genes, the expression of which differed between patients with polymyalgia rheumatica (PMR) and non-PMR control subjects before prednisolone treatment and which also responded to treatment with prednisolone in a given direction only in PMR patients. The clusters and the enrichment scores (the geometric means of the EASE scores [24] of all terms in the cluster) were derived using the Database for Annotation, Visualization and Integrated Discovery (DAVID) tool [23]. Green squares denote that the gene/term association has been positively reported; *black* squares denote that the gene/term association has not yet been reported. **a** Cluster with an overall theme of regulation of transcription and with an enrichment score of 1.59. **b** Cluster with an overall theme of translation/protein biosynthesis and with an enrichment score of 0.63

In this subset, out of a total of 8 DAVID-identified clusters, the clusters with the highest enrichment scores (Fig. 4) comprised genes with transcription regulation (2 clusters, enrichment scores 1.59 and 1.17 [data not shown) and protein translation/biosynthesis (2 clusters, enrichment score 0.63 and 0.59 [data not shown) properties.

### qRT-PCR

**Fig. 5:**
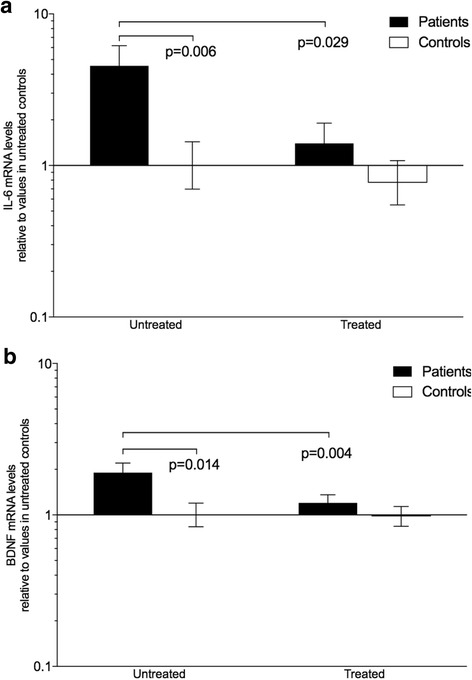
Muscle (**a**) interleukin 6 (IL-6) and (**b**) brain-derived neurotrophic growth factor (BDNF) mRNA levels normalized to the mRNA levels of the gene encoding ribosomal protein, large P0 (RPLP0; arbitrary units), in patients with polymyalgia rheumatica (PMR, *n* = 9) and non-PMR control subjects (*n* = 9) before and after treatment with prednisolone (20 mg/day) for 14 days. Values are relative to untreated controls (=1.0) and shown on a logarithmic scale. Data are geometric mean and errors bars SEM

To validate the levels found using microarrays, the expression of some of the genes were measured using qRT-PCR (Tables 5 and 6, and Fig. 5).

Nine genes that fulfilled criterion 1 or criterion 2 according to microarray analysis were examined with qRT-PCR (Table 5 and Fig. 5b); 8 of the 9 genes were always regulated in the same direction as found using microarrays. However, for the comparison of patients and controls before treatment (criterion 1), the expression fold differences of 5 genes (COL5A1, MARK4, MTFP1, PRSS23, and TRFC), which were statistically significant in the microarray analysis, did not reach significance using qRT-PCR (*p* > 0.05). For the treated vs untreated patients comparison (criterion 2), the fold changes for NPM1, PRSS23 and TUBD1 were significant in the microarray but not in the qRT-PCR, whereas the fold change for MARK4 was significant only in qRT-PCR analysis. The fold changes for COL5A1 and TRFC were not statistically significant (*p* > 0.05) in the microarray nor in the qRT-PCR analysis.

Moreover, the expression levels of 5 genes (Table 5) of potential interest in PMR that did not differ in the microarray analysis were measured using qRT-PCR. Expression levels of IL-6 (Fig. 5a), which did not differ in the microarray experiments (FD and FC < 1.1), markedly differed both between untreated patients and controls (FD 4.54, *p* < 0.05) and between patients before and after treatment (FC —3.25, *p* < 0.05) using qRT-PCR (Table 5). The remaining four genes were found to differ neither between untreated patients and controls nor between patients before and after treatment with either method.

## Discussion

In the present study, the gene expression in skeletal muscle was measured for the first time in patients with PMR and in non-PMR, matched controls subjects before and after brief, symptom-relieving prednisolone treatment using DNA microarrays. Microarray findings were supplemented by testing of the expression levels of selected genes with qRT-PCR, which was also used to accurately measure expression levels of genes of particular interest. In all subjects, biopsies were obtained from the trapezius muscle. Before treatment, patients had marked clinical symptoms, including trapezius myalgia and tenderness, as well as elevated ESR and levels of CRP; upon treatment, paraclinical parameters had normalized and clinical symptoms had disappeared.

Subjects were studied in 2008; thus, we were not able to use the most recent PMR criteria, which were published in 2012 [17]. However, the latter criteria are still provisional and awaiting further validation, and, in the most recent reviews of PMR, the Chuang criteria are mentioned on par with the newer provisional criteria [2, 3, 8, 17]. The two criteria sets are very similar; however, the fact that the demand for a high ESR is stricter in the Chuang criteria implies that the patients in the present study would also be accepted with the new criteria.

A total of 565 genes were differentially expressed across all groups. In general, when measured by microarray, fold differences and fold changes in expression were modest, ranging from 1.2 (cut-off value) to 1.4 for most genes. Despite the relatively modest differences in gene expression levels, gene function analysis indicated that even these small differences may have a pathophysiological and phenotypic impact in PMR. A few genes were regulated more markedly, with fold differences and changes in the range of 2 to 4. In the microarray measurements, none of the genes that usually are associated with PMR [12], for example genes encoding proteins involved in inflammation, e.g. IL-6, were differentially expressed in symptom-yielding muscle tissue. However, using the more sensitive qRT-PCR technique, the expression of the IL6 gene showed marked differences between groups, being up-regulated in untreated patients and down-regulated after prednisolone treatment (Fig. 5). This finding is in line with a previous microdialysis study that indicated a local production of IL-6 in symptom-yielding muscles in patients with PMR, and normalization with prednisolone treatment [5]. Furthermore, several studies [1, 4, 5, 25] have found that plasma IL-6 is highly elevated in PMR. In line with a key role in the pathophysiology of the disease, IL-6 blockade has recently in an open-label study been shown to be an effective treatment for newly diagnosed PMR [26].

### Genes differentially expressed in untreated PMR patients vs controls

The applied study design allowed for 3 important comparisons. Firstly, by comparing expressions levels in untreated patients and control subjects, 78 genes of possible central importance for the phenotype of PMR were identified.

Although the enrichment scores, which are proportional to the extent to which the cluster is represented in the gene set (here 78 genes), were modest within this subset of genes, functional clustering analysis identified several clusters of genes, many of which were associated with protein translation and biosynthesis. Other identified clusters included regulation of transcription, cellular and nuclear protein transport, and rearrangement of the cytoskeleton; the latter process was also represented in a gene cluster that involved SH-3-domain-binding properties, which are associated with cytoskeletal elements and signaling proteins.

The identification of clusters associated with protein translation, biosynthesis and transport may suggest that PMR is associated with abnormal protein metabolism in muscle. It might be speculated that inflammation and immobilization, which induce negative protein balance in many chronic diseases, accounted for these findings. However, in the protein translation and biosynthesis clusters, more genes were up-regulated rather than down-regulated in patients versus controls in the present cohort. Furthermore, indicating a minor role of inactivity in the present study, the number of genes in muscle influenced by PMR was small compared to findings in response to inactivity per se [27].

Another finding that may possibly contribute to the muscle complaints, primarily the muscle stiffness, experienced by PMR patients [28] is that proteins involved in organizing the cytoskeleton, including tubulin delta 1 (TUBD1; similar findings with microarrays and with qRT-PCR) and microtubule affinity-regulating kinase 4 (MARK4; similar differences in microarray and qRT-PCR, but only significant in the former), were up-regulated in patients before prednisolone treatment (Tables 2 and 5) [28].

Another interesting gene in this subset was the gene encoding brain-derived neurotrophic growth factor (BDNF). This neurotrophic growth factor was markedly upregulated in patients before treatment as determined by both microarray and qRT-PCR (Tables 2 and 5, Fig. 5). While BDNF traditionally is associated with diseases such as Alzheimer’s and mood disorders [29], studies have shown that BDNF is also expressed in satellite cells surrounding skeletal muscle cells, and, based on studies in rats, a role for BDNF in maintaining the satellite cell population has been suggested [30]. We have previously shown that PMR is associated with high intramuscular levels of proinflammatory cytokines [5], and it might be speculated that in untreated PMR, BDNF is upregulated to counter the muscle damage resulting from the inflammatory processes as well as the muscle degeneration resulting from the reduced physical activity level of PMR patients.

Finally, the transferrin receptor/CD71 (TFRC) gene was down-regulated 3 fold in patients before treatment. The transferrin receptor protein is involved in the transport of iron into cells, it is required for erythrocyte development, and it is associated with diseases such as iron deficiency, anemia, and chronic disease in general. It has been suggested that low levels of soluble transferrin receptors reflect adaptation to iron deficiency and/or inhibition of iron resorption [31]. It is conceivable that in this group of patients, TFRC is down-regulated due to the chronic inflammatory disease burden associated with PMR. While intramyocellular iron deficiency may ensue, it is not likely that the muscular down regulation of TFRC was secondary to systemic iron deficiency. This is so because none of the subjects exhibited anemia. Other studies have identified that PMR is associated with antibodies against ferritin [32, 33]. Taken together, this suggests that iron metabolism and the function of proteins that rely on iron-binding may be influenced in PMR.

### Genes responding to prednisolone in PMR patients

The phenotype of PMR in this and other studies [1, 5, 15, 34] profoundly responds to treatment with glucocorticoids, indicating that important information about the pathophysiology of the disease can be achieved by studying the gene expression before and after prednisolone treatment. Moreover, if studying only untreated subjects, it is conceivable that, due to sampling errors, including unrecognized impacts of e.g. diurnal gene expression variations between patients and controls, discovery of all genes relevant to the pathophysiology of PMR would not be achieved. For these reasons, comparison of expression levels before and after symptom eliminating prednisolone treatment in patients was also used for the identification of genes with importance for PMR. The number of genes that responded to treatment in a given direction only in patients was 131. Indicating that these genes were, in fact, involved in the pathophysiology of PMR, of the 131 mentioned genes that responded to treatment in patients, only 2 also responded in controls subjects, and they did so in the direction opposite to that seen in the patients. Genes responding in the same direction to prednisolone in both patients and controls were not emphasized, because it is likely that the response reflected a general effect of glucocorticoids of no importance for the pathophysiology of PMR.

The functional clusters in this subset of genes included genes involved in the organization of the cytoskeleton and genes relevant for the extracellular matrix. In this context, it is of note that both TUBD1 and MARK4 were down-regulated by prednisolone, the fold changes being significant in microarray and qRT-PCR, respectively (Table 5). The fact that such genes respond to prednisolone treatment in patients with PMR is in line with the hypothesis that muscle stiffness may be due to abnormal expression of cytoskeleton-related genes. Correspondingly, clinical remission, including abolishment of muscle stiffness, happened in parallel with or due to normalization of expression of such genes.

### Genes differentially expressed in untreated PMR patients vs controls and also responding to prednisolone in patients

The strongest evidence in favor of a pathogenic role of a given gene would be that its expression differed between untreated patients and controls, and, furthermore, changed with prednisolone treatment in the former. The number of such genes was 44 in the present cohort. Strongly indicating that these genes do in fact play a role in PMR, the response to prednisolone of all but one of the 44 genes counteracted the difference in gene expression between untreated patients and controls. In this group of genes, the predominant biological functions appeared to be regulation of transcription as well as protein translation/biosynthesis.

The finding that the expression of some genes differed between untreated patients and controls while not responding to prednisolone treatment in patients may indicate that clinical remission may be achieved even though the underlying disease mechanisms are not completely resolved or that not all differences in gene expression may be of importance for clinical symptoms. As a limitation of the present study, it should be noted, however, that while all patients achieved clinical remission during the relatively brief 14-day treatment period, some genes might respond to long-term treatment only. Conversely, it is also interesting to note that in the untreated patients, some genes, the expression of which did not differ from that of controls, were, nevertheless, selectively influenced by prednisolone. It may be that in the patients the processes regulated by these genes were impaired by other, non-genetic factors that possibly also resulted in increased sensitivity to prednisolone. If so, the condition would be ameliorated by a prednisolone-induced effect on these genes.

## Conclusions

This study is the first to demonstrate changes in the gene expression in skeletal muscle in PMR. The study has identified a number of genes that may play a role in the pathophysiology of PMR. Moreover, we show that the expression of the IL6 gene is upregulated in muscle in PMR, a finding that adds to the substantial body of evidence that this cytokine is central to the disease. Follow-up studies are needed to elucidate the exact pathophysiological relevance of the identified genes; however, it appears that many of the genes are involved in the regulation of protein biosynthesis, which may suggest that abnormal protein metabolism is a disease mechanism in PMR. Effects of prednisolone on genes involved in the organization of the cytoskeleton and the intracellular matrix in PMR patients may contribute to the amelioration, seen in response to treatment, of the muscle stiffness.

## Abbreviations

CRP: C-reactive protein
ESR: Erythrocyte sedimentation rate
GC: Glucocorticoid
IL: Interleukin
PMR: Polymyalgia rheumatica
qRT-PCR: Quantitative real-time polymerase chain reaction
